# Multidimensional modeling and stratification of nutritional risk in non-small cell lung cancer based on artificial intelligence–derived CT body composition phenotypes

**DOI:** 10.3389/fnut.2026.1842431

**Published:** 2026-08-20

**Authors:** Dianhui Xiu, Yanjing Wang, Xiaosong Sun, Kailiang Cheng, Zhen Ye, Lin Liu, Min Cheng

**Affiliations:** 1 Department of Radiology, China-Japan Union Hospital, Jilin University, Changchun, China; 2 Department Head and Neck Surgery, Jilin Cancer Hospital, Changchun, Jilin, China

**Keywords:** artificial intelligence, body composition, chest CT, immune-inflammation, non-small cell lung cancer, nutritional risk, risk stratification

## Abstract

**Objective:**

To develop and temporally validate a multidimensional nutritional risk model integrating artificial intelligence (AI)–derived CT body composition phenotypes for patients with non-small cell lung cancer (NSCLC).

**Methods:**

This single-center retrospective cohort included 656 patients with pathologically confirmed NSCLC, including 432 in the development cohort and 224 in the temporal validation cohort. Baseline chest CT, clinical-nutritional variables, and immune-inflammatory markers were collected before first-line treatment. AI-based automated body composition analysis was used to extract CT phenotypes. The primary outcome was a 90-day nutrition-related adverse clinical trajectory. Clinical-nutritional, imaging, and integrated models were developed and compared.

**Results:**

The incidence of short-term nutrition-related adverse clinical trajectories was 34.3% in the development cohort and 32.6% in the validation cohort. Percentage weight loss, prognostic nutritional index, systemic immune-inflammation index, skeletal muscle index, and intermuscular adipose tissue volume were independent predictors. The integrated model achieved the best performance, with AUCs of 0.842 and 0.816 in the development and validation cohorts, respectively, and showed superior calibration and clinical utility. Higher risk scores were also associated with worse overall survival and progression-free survival.

**Conclusion:**

Integrating AI-derived CT body composition phenotypes with nutritional and inflammatory indicators improved nutritional risk stratification in NSCLC and may support earlier risk-adapted nutritional management.

## 1 Introduction

Lung cancer remains one of the most burdensome malignancies worldwide. In 2022, nearly 2.5 million new cases were diagnosed globally, accounting for 12.4% of all malignant tumors and approximately 1.8 million deaths, making it the leading cause of cancer-related mortality (1). In China, lung cancer remained the most commonly diagnosed malignancy in 2024 (2). Although advances in screening, surgery, radiotherapy, systemic therapies, and immunotherapy have improved outcomes for some patients, population-level long-term survival gains remain limited (3). Non-small cell lung cancer (NSCLC) accounts for about 85% of all lung cancers and is the focus of precision treatment (4). However, current clinical decision-making centers largely on tumor stage and molecular features, with insufficient attention to host-level heterogeneity. Key dimensions such as nutritional reserve, body composition, and systemic inflammation have not been systematically integrated, limiting understanding of treatment tolerance and outcome heterogeneity.

Nutritional derangement is a major basis of clinical vulnerability in NSCLC and influences outcomes even in the immunotherapy era. A systematic review found that nutrition-related adverse events remain common in patients receiving immune checkpoint inhibitors, manifesting as reduced oral intake, gastrointestinal toxicity, and weight loss (5). A multicenter study of 3,634 lung cancer patients reported that approximately 42% experienced nutrition impact symptoms, which were closely associated with severe malnutrition, cachexia, poorer quality of life, and shorter overall survival (6). The NUTIMMUNO study showed that patients with early nutritional improvement during immunotherapy had significantly better tumor responses (7). Nevertheless, even in advanced NSCLC with anorexia, randomized evidence indicates that effective, standardized intervention strategies are still lacking (8). Thus, identifying high nutritional risk before or early during treatment is clinically important.

Tools such as NRS-2002 and GLIM form the main framework for oncologic nutritional assessment. GLIM-defined malnutrition is associated with adverse postoperative outcomes in primary lung cancer (9), and both NRS-2002 and GLIM can stratify overall survival in patients receiving immune checkpoint inhibitors (10). However, their application in NSCLC has limitations. Phenotypic assessment still relies mainly on weight change, BMI, and clinical history, making it difficult to detect patients with substantial muscle depletion or abnormal fat redistribution despite stable weight. A recent meta-analysis noted inherent shortcomings in using BMI as a surrogate for nutritional abnormality within GLIM criteria (11). Moreover, a systematic review of cancer cachexia endpoints showed that CT-based body composition quantification remains underused, with limited measurement consistency (12). Thus, conventional nutrition screening and static anthropometric indicators alone cannot fully capture the nutritional reserve and host vulnerability in NSCLC.

CT-based body composition analysis more directly characterizes the metabolic remodeling of the tumor host through skeletal muscle and adipose tissue. In advanced NSCLC patients receiving immunotherapy, skeletal muscle loss during treatment is associated with significantly poorer outcomes, and changes in subcutaneous fat density are also closely linked to survival, indicating that muscle and fat provide complementary information on physiological reserve and metabolic status (13). A multicenter study found that baseline body composition abnormalities were significantly associated with tumor response and clinical outcomes in first-line pembrolizumab-treated advanced NSCLC (14). Higher subcutaneous adipose tissue radiodensity has been independently associated with increased tumor metabolic activity and shorter overall survival, suggesting that qualitative adipose changes reflect tumor-driven systemic metabolic disturbance (15). Importantly, real-world data show that body weight and body composition changes during first-line therapy do not fully coincide; some patients exhibit substantial muscle loss or fat redistribution despite minimal weight change, confirming that BMI and weight cannot substitute for body composition information (16). Therefore, a refined nutritional phenotype based on muscle reserve, muscle quality, and fat distribution may be valuable for identifying latent nutritional vulnerability in NSCLC.

Nutritional risk in NSCLC is also tightly linked to systemic immune-inflammatory status. In a study of 500 advanced NSCLC patients treated with immunotherapy, multiple pretreatment nutritional and inflammatory biomarkers were significantly associated with overall survival, with CAR and PNI showing the most stable associations (17). In stage IV NSCLC treated with immunotherapy, peripheral blood markers such as LAR, PIV, and PNI helped construct predictive models to identify patients more likely to benefit (18). In neoadjuvant immunochemotherapy for locally advanced NSCLC, dynamic changes in peripheral inflammatory markers combined with albumin and PNI predicted major pathologic response and event-free survival, indicating that immune-inflammatory and nutritional status are dynamic host signals (19). Inflammatory-nutritional indices derived from tumor metabolic activity, including FAR and ALI, have shown stable prognostic stratification across NSCLC cohorts (20–22). However, most previous studies have been limited to single hematologic markers or linear combinations, without systematic integration with imaging-derived body composition phenotypes, thus insufficiently characterizing nutritional vulnerability from a multidimensional perspective.

Artificial intelligence provides new methodological support for multidimensional nutritional risk assessment. Automated segmentation of skeletal muscle and adipose tissue on CT or MRI has become an important direction in body composition quantification, improving standardization, reproducibility, and scalability (23). AI-driven three-dimensional thoracic body composition quantification can substantially enhance mortality prediction (24), and segmentation-volume optimization may enable more accurate estimation of total muscle burden (25). Deep-learning harmonization methods can reduce measurement bias across scanning protocols (26). In oncology, AI-based three-dimensional CT body composition models improve prediction of dose-limiting chemotherapy toxicity and outperform traditional single-slice strategies (27).

Thus, opportunistic extraction of body composition phenotypes from routine chest CT in patients with NSCLC, combined with clinical nutritional indicators and immune-inflammatory information to build a multidimensional risk-assessment framework, is methodologically sound and clinically translatable. The aim of this study was therefore to develop and validate a multidimensional nutritional risk prediction model based on AI-derived CT body composition phenotypes for refined risk stratification in NSCLC, and to systematically evaluate its ability to identify short-term nutrition-related adverse clinical trajectories and long-term prognosis.

## 2 Methods

### 2.1 Study design and reporting framework

This was a single-center retrospective cohort study designed to develop and validate a multidimensional risk-assessment model based on automated body composition analysis from chest CT for nutritional risk stratification in patients with NSCLC, and to evaluate its association with clinical prognosis. Using real-world clinical data, the study focused on the potential value and clinical relevance of body composition imaging phenotypes extracted from routine chest CT for identifying nutritional risk.

The design and reporting of this study followed internationally recognized methodological standards. The observational component was conducted in accordance with the Strengthening the Reporting of Observational Studies in Epidemiology (STROBE) statement. The development and validation of the prediction model followed the Transparent Reporting of a multivariable prediction model for Individual Prognosis Or Diagnosis–Artificial Intelligence (TRIPOD+AI) guidance. Imaging-AI-related content was reported with reference to the Checklist for Artificial Intelligence in Medical Imaging (CLAIM) framework to ensure transparency and reproducibility in data sources, model application, and outcome evaluation.

In this study, the AI-based body composition analysis system was defined as an imaging phenotype extraction tool, with the primary role of enabling automated and standardized acquisition of body composition information from chest CT. The main analytical target of the study was the nutritional risk prediction model constructed by combining imaging-derived body composition features with clinical nutritional and immune-inflammatory information. Accordingly, the present report focused on the risk stratification capability and clinical validity of multidimensional information integration, rather than on the development or performance optimization of the segmentation algorithm itself. On this methodological basis, the following sections describe the study population, endpoint definitions, variable construction, imaging analysis workflow, and model development and validation strategy in a structured manner to ensure analytical rigor.

### 2.2 Study population and cohort construction

We consecutively identified patients with pathologically confirmed NSCLC who were seen at Department of Radiology, China-Japan Union Hospital of Jilin University between January 1, 2022 and December 31, 2025. All cases were retrieved from the hospital electronic medical record system and picture archiving and communication system (PACS), with cross-checking against the laboratory information system.

The inclusion criteria were as follows: (1) presentation to our center during the study period and age ≥18 years; (2) histopathologic or cytopathologic confirmation of NSCLC, including lung adenocarcinoma, lung squamous cell carcinoma, and other NSCLC subtypes meeting World Health Organization classification criteria; (3) completion of baseline chest CT before initiation of initial anticancer treatment, with an interval of no more than 14 days between CT and treatment initiation; initial anticancer treatment included surgery, chemotherapy, radiotherapy, concurrent chemoradiotherapy, targeted therapy, and immunotherapy; (4) availability of complete contemporaneous hematologic and biochemical test results within 3 days before or after baseline CT to ensure that laboratory indices objectively reflected inflammatory, immune, and nutritional status at the imaging time point; (5) availability of traceable baseline anthropometric and nutrition-screening data, including height, weight, and baseline nutritional risk screening information, sufficient for subsequent variable definition and analysis; (6) availability of complete baseline clinical data, including demographic information, tumor pathological features, staging information, and initial treatment details; and (7) availability of follow-up data sufficient for endpoint adjudication and prognostic analyses.

The exclusion criteria were as follows: (1) concomitant active primary malignancy other than NSCLC, to avoid confounding related to cancer-associated catabolism, treatment exposure, or prognosis from other tumors; (2) baseline chest CT not meeting the predefined temporal requirements, including CT performed after initiation of any anticancer treatment or performed before treatment but more than 14 days prior to treatment initiation, to ensure that imaging reflected true pretreatment baseline status; (3) CT image quality not meeting predefined analysis requirements, including but not limited to severe motion artifact, inadequate breath-hold, abnormal reconstruction slice thickness or reconstruction parameters, incomplete anatomic coverage, or missing DICOM data precluding standardized body composition quantification; (4) anatomical or technical factors substantially affecting the accuracy of automated thoracic body composition segmentation or quantification, including large pleural effusions, marked thoracic deformity, extensive postoperative changes, major metallic artifacts involving the chest wall or mediastinum, or other conditions judged by the investigators to distort body composition measurement; (5) missing key clinical variables, especially core data required for the main exposure, covariates, or stratification factors, that could not be recovered from the medical record; (6) missing key outcome data or insufficient follow-up precluding adjudication of the primary endpoint or evaluation of prognostic outcomes; and (7) duplicate visits or duplicate imaging records, in which case only the baseline assessment closest to pretreatment and with the most complete data was retained.

Using these criteria, all potentially eligible cases were screened individually. A total of 1,128 patients met the pathological diagnosis criterion, of whom 472 were excluded for not meeting the study requirements. The main reasons for exclusion were baseline CT outside the predefined window (*n* = 154), missing laboratory data (*n* = 83), missing height, weight, or nutritional screening data (*n* = 71), inadequate image quality (*n* = 49), pathological type not meeting inclusion criteria (*n* = 62), prior or concurrent additional primary malignancy (*n* = 15), structural or technical factors affecting body composition quantification (*n* = 10), and missing follow-up data (*n* = 28).

**Figure 1:**
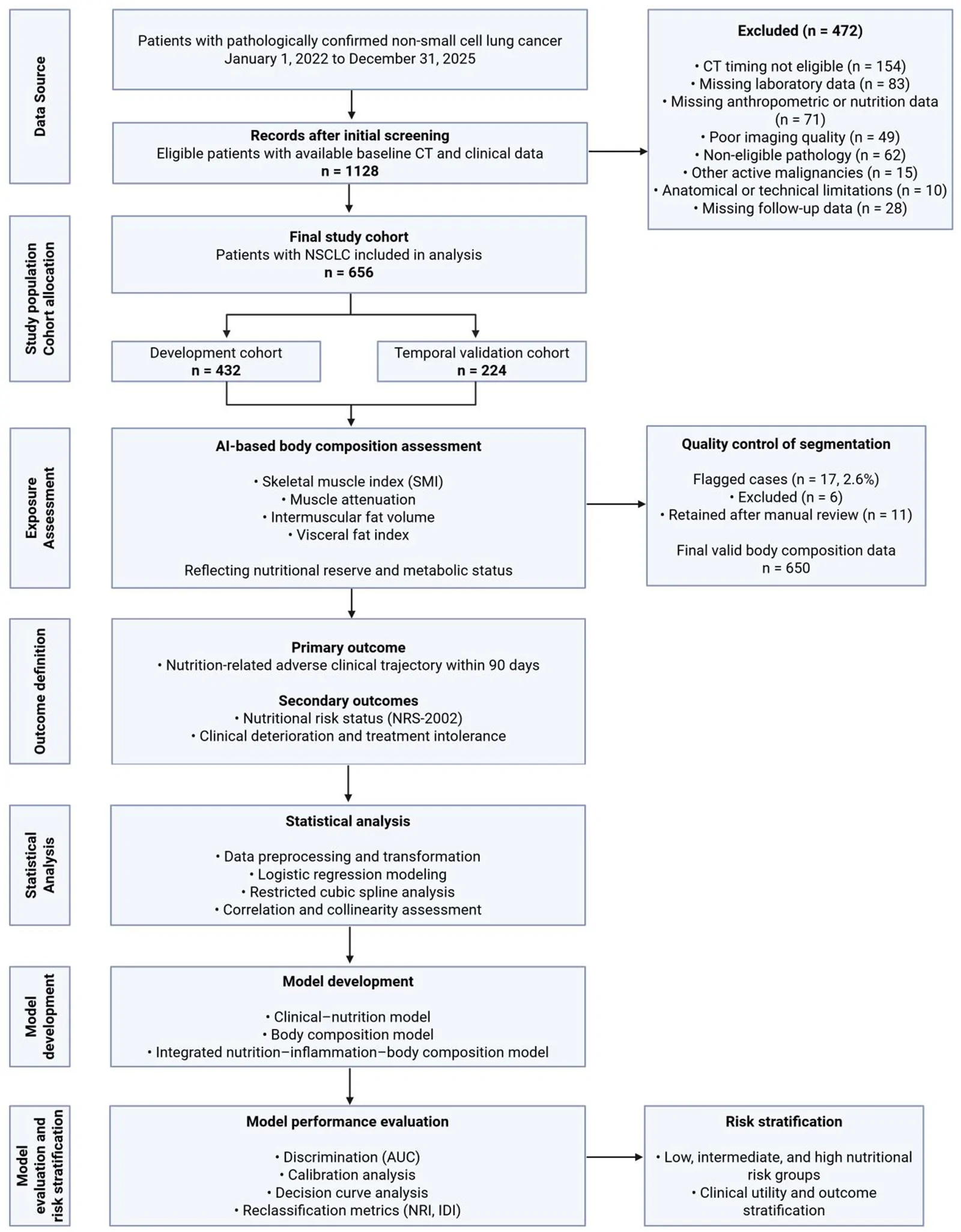
Study flowchart and CT-based framework for nutrition-related body composition analysis.

Ultimately, 656 patients were included in the analytic cohort. To improve external transportability and avoid information leakage associated with random splitting, patients were divided chronologically according to date of presentation. Patients enrolled between January 1, 2022 and December 31, 2023 constituted the development cohort (*n* = 432), which was used for model building and internal validation, whereas those enrolled between January 1, 2024 and December 31, 2025 constituted the independent temporal validation cohort (*n* = 224), which was used for external temporal validation of model performance. To contextualize the temporal validation, we systematically reviewed institutional practices during the study period. Between the development and validation eras, no major changes occurred in first-line treatment guidelines, nutritional support protocols, or supportive care pathways at our institution, except for the gradual adoption of immune checkpoint inhibitors in adjuvant settings from mid-2024 onward, which affected a small proportion of patients (6.3% in the validation cohort vs. 4.2% in the development cohort, *P* = 0.241) and was adjusted for in multivariable analyses. The availability and criteria for nutritional consultation remained unchanged throughout the study period. The patient selection process is shown in Figure 1.

### 2.3 Study endpoints and reference standards

#### 2.3.1 Primary endpoint: short-term nutrition-related adverse clinical trajectory

The primary endpoint of this study was a short-term nutrition-related adverse clinical trajectory occurring within 90 days after baseline assessment, chosen to enhance the real-world risk-identification value and translational utility of the model. A single diagnosis of malnutrition was not used as the sole primary endpoint because the clinical significance of nutritional risk stratification extends beyond static identification of whether a patient has crossed a diagnostic threshold. More importantly, it involves early identification of patients with a clinically vulnerable phenotype who are more likely to experience poorer treatment tolerance, worsening disease course, and greater healthcare utilization.

Accordingly, a short-term nutrition-related adverse clinical trajectory was defined as the occurrence of any of the following within 90 days from the date of baseline chest CT: (1) delay of anticancer treatment by at least 2 weeks, dose reduction, or unplanned interruption of therapy; (2) unplanned hospitalization related to treatment toxicity or deterioration in general condition; (3) postoperative complications of Clavien–Dindo grade II or higher in patients undergoing surgery; (4) treatment-related adverse events of grade 3 or higher according to Common Terminology Criteria for Adverse Events, version 5.0 (CTCAE 5.0); or (5) all-cause death within 90 days after baseline assessment. Given the inherent heterogeneity of this composite endpoint, we prespecified that the occurrence rates of each individual component would be reported separately, and that sensitivity analyses using each component as the sole outcome would be performed to assess the consistency of model performance across different clinical event types.

The rationale for this composite endpoint was to extend the focus of the study from conventional nutritional status classification to the identification of nutritional vulnerability and its short-term clinical consequences. This endpoint framework more comprehensively reflects the integrated impact of nutritional imbalance on functional reserve, treatment tolerance, and near-term clinical outcomes during treatment delivery, thereby improving the model's ability to inform the timing of clinical intervention. Compared with using a single nutrition screening tool or malnutrition diagnosis as the outcome, this endpoint is better aligned with the practical goals of oncologic nutritional management, namely early identification, forward intervention, and dynamic stratification, and is also better suited to demonstrate the incremental value of imaging-derived body composition information in clinical risk warning.

#### 2.3.2 Secondary endpoints: standardized nutritional risk and malnutrition status

To ensure that the study findings would be methodologically rigorous, clinically interpretable, and comparable with the nutrition literature, standardized nutritional risk and malnutrition status were further specified as key secondary endpoints. Nutritional risk was assessed using Nutritional Risk Screening 2002 (NRS-2002), with a score of ≥3 defining high nutritional risk. Malnutrition was determined using the Global Leadership Initiative on Malnutrition (GLIM) framework.

Under the GLIM framework, the diagnosis of malnutrition requires at least one phenotypic criterion and at least one etiologic criterion. Phenotypic criteria include involuntary weight loss, low body mass index, and low muscle mass, whereas etiologic criteria include reduced food intake or assimilation and inflammation or disease burden. Given the broad international acceptance of GLIM in clinical nutrition research and its established role in identifying cancer-related malnutrition, its use as a key secondary endpoint facilitates alignment of the present findings with the existing evidence base.

The purpose of this strategy was to preserve, as far as possible, the relative independence between the study endpoint and the investigational imaging features, thereby improving the rigor of model performance evaluation and the credibility of result interpretation. Although low muscle mass and malnutrition are closely related in patients with cancer, they are not completely overlapping in biological meaning or clinical manifestation. Maintaining a degree of separation between them is therefore more conducive to revealing the independent contribution and potential clinical significance of imaging-detected body composition abnormalities in nutritional risk stratification.

This approach does not deny the importance of low muscle mass within the GLIM framework. Rather, it reflects a methodological decision specific to prediction-model research to prioritize relative independence between label construction and investigational imaging predictors. Compared with directly incorporating imaging-derived muscle mass into GLIM according to the original operational pathway, the present study placed greater emphasis on avoiding circular definitions, information overlap, and performance inflation during model training. In essence, this strategy represents a cautious balance between completeness of nutritional definitions and independence of predictive evaluation, with the aim of improving the authenticity, rigor, and interpretability of model assessment.

It should be emphasized that GLIM-defined malnutrition was not used as the dependent variable for the primary prediction model. Rather, GLIM served exclusively as a nutritional construct anchor for descriptive characterization of the study population and for subgroup analyses. The primary model development and all main performance evaluations were based on the composite 90-day nutrition-related adverse clinical trajectory as the outcome. This design choice was made specifically to avoid label leakage—i.e., to prevent the circularity that would arise if imaging-derived body composition parameters were used both to define the GLIM malnutrition label (via the low muscle mass phenotypic criterion) and to predict that same label. By decoupling the model-building outcome from the GLIM definition, we preserved the relative independence between the investigational imaging predictors and the endpoint label, thereby reducing the risk of performance inflation and improving the credibility of the model evaluation.

#### 2.3.3 Long-term prognostic outcomes

To further examine the long-term clinical significance of nutritional risk stratification, overall survival (OS) and progression-free survival (PFS) were defined as exploratory outcomes. OS was defined as the time from the date of baseline chest CT to all-cause death or last follow-up. PFS was defined as the time from baseline chest CT to radiologic or clinical disease progression, death, or last follow-up.

The purpose of including these outcomes was not to develop a separate survival prediction model, but to test whether the high-risk phenotype identified by nutritional risk stratification also indicated poorer long-term prognosis, thereby evaluating its potential utility beyond short-term management. If short-term nutrition-related risk were also stably associated with long-term survival outcomes, this would support the notion that the proposed framework has not only near-term event-warning capacity but also the potential to reflect broader disease vulnerability and host reserve. In this sense, the long-term prognostic endpoints served primarily as an external clinical validity assessment to further evaluate the longitudinal discriminatory capacity and translational promise of the risk stratification system.

### 2.4 Collection of clinical, nutritional, and immune-inflammatory variables

All baseline data were retrospectively extracted from the electronic medical record system, laboratory information system, and PACS, and were cross-checked by two investigators according to a predefined variable dictionary. To ensure temporal consistency across different variable types, all clinical, nutritional, and laboratory indices were collected with reference to the baseline chest CT time point. If multiple measurements were available within the same time window, the value closest to the CT examination was selected for analysis.

Clinical variables included age, sex, smoking history, Eastern Cooperative Oncology Group performance status (ECOG PS), comorbidities, pathological subtype, tumor stage, and initial treatment modality. Tumor staging was determined according to the relevant TNM staging system in use during the study period. Initial treatment modalities included surgery, chemotherapy, radiotherapy, concurrent chemoradiotherapy, molecular targeted therapy, and immunotherapy. These variables were used to characterize baseline demographics, tumor biological features, and treatment exposure, and to control for potential confounding in subsequent analyses.

Nutritional variables included height, weight, body mass index, percentage of involuntary weight loss over the previous 6 months, serum albumin, prealbumin, NRS-2002 score, and prognostic nutritional index (PNI). BMI was calculated as weight divided by height squared. Percentage involuntary weight loss over 6 months was defined relative to a prior stable body weight. PNI was calculated as: PNI = albumin (g/L) + 5 × lymphocyte count (× 10^9^/L). Together, these variables reflected baseline nutritional status from the perspectives of anthropometry, dynamic weight change, and protein nutritional status, and served as the reference framework for conventional nutritional assessment.

Immune-inflammatory variables were obtained from the peripheral blood test performed closest to the baseline CT examination within ±3 days, including neutrophil count, lymphocyte count, monocyte count, platelet count, and C-reactive protein. Derived indices reflecting host inflammatory and immune status were further calculated, including neutrophil-to-lymphocyte ratio (NLR), platelet-to-lymphocyte ratio (PLR), lymphocyte-to-monocyte ratio (LMR), and systemic immune-inflammation index (SII), where SII = platelet count × neutrophil count/lymphocyte count.

These immune-inflammatory indices were included to capture tumor-related systemic inflammatory response and host immune status, thereby complementing the multidimensional characterization of nutritional abnormality and its clinical consequences. Based on this variable collection framework, all clinical variables, nutritional variables, immune-inflammatory indices, and imaging parameters generated by AI-based body composition analysis were treated as candidate predictors. Before model development, these variables underwent standardized preprocessing and feature engineering. By integrating candidate variables from different informational layers, a series of hierarchical prediction models was built to systematically evaluate the independent roles and potential synergistic effects of each variable domain in nutritional risk identification.

### 2.5 CT acquisition protocol and image quality control

All imaging studies included in this analysis were chest CT examinations acquired as part of routine clinical care. Original images were exported from the institutional PACS in Digital Imaging and Communications in Medicine (DICOM) format. For all included examinations, relevant scan parameters were recorded, including scanner model, tube voltage, tube current mode, slice thickness, reconstruction algorithm or kernel, contrast-enhancement status, and scan coverage. To minimize technical heterogeneity introduced by varying scan conditions, only examinations with complete thoracic coverage and slice thickness meeting predefined analysis requirements were included.

Before entering the subsequent image-analysis workflow, all CT scans underwent standardized quality review. Quality control was performed independently by two investigators experienced in thoracic image interpretation and focused mainly on completeness of anatomic coverage, the presence of obvious motion or respiratory artifact, the presence of metallic artifacts affecting interpretation and quantification, and whether reconstruction parameters met predefined technical standards. Any image with uncertain quality was adjudicated by a senior thoracic radiologist, whose decision was final. Images of insufficient quality for standardized body composition analysis were excluded from the final study cohort.

If more than one chest CT scan met the basic eligibility criteria within the predefined time window for a given patient, only the scan closest to pretreatment and with the most complete image quality was retained as the baseline examination. This approach was intended to ensure that the included imaging most accurately reflected pretreatment host status and to reduce information bias arising from inconsistent selection among repeated examinations.

This section is presented separately in order to improve transparency and reproducibility with respect to imaging data sources, control of technical heterogeneity, and sample inclusion procedures, thereby ensuring that subsequent body composition quantification was based on a consistent, reliable, and traceable imaging foundation.

### 2.6 AI-based chest CT body composition analysis workflow

#### 2.6.1 Role definition of the AI body composition analysis tool

A deep-learning-based AI system was used to perform automated identification and quantification of body composition on chest CT images. In the present study, this system was defined as an imaging phenotype extraction tool whose role was to enable automated and standardized acquisition of body composition information, rather than a research object requiring retraining or optimization.

The model used had been previously developed and validated on independent datasets, and it was applied directly to all included imaging data with frozen parameters, without retraining or structural modification. Information regarding its training data, network architecture, and prior validation performance had been reported in previous work. By using fixed model parameters, the risk of overfitting associated with retraining on the current study data was avoided, thereby ensuring the independence and stability of the body composition measurement process.

Within the methodological framework of this study, the AI system functioned as part of a standardized measurement pipeline, and its outputs were used for subsequent clinical model development and validation. This setup is consistent with the prevailing methodological approach in body composition imaging research, in which the automated segmentation algorithm is treated as one component of the measurement pipeline, while the primary research focus is placed on the incremental clinical value of imaging-derived phenotypes.

#### 2.6.2 Image preprocessing and automated segmentation

Before AI analysis, all included CT images underwent uniform preprocessing. Preprocessing steps included voxel resampling to harmonize spatial resolution, intensity normalization to reduce the influence of scan-condition differences, and standardization of anatomic coverage to ensure consistency of the region analyzed.

After preprocessing, images were input into the deep-learning model for automatic identification of thoracic anatomic structures and segmentation of body composition tissues. The segmented components mainly included skeletal muscle, subcutaneous adipose tissue, visceral adipose tissue, and intermuscular adipose tissue. Depending on the study design, body composition parameters could be extracted either as cross-sectional area measures from predefined thoracic vertebral levels or as volume-related measures from the three-dimensional thoracic region.

Because single-slice two-dimensional measurements are susceptible to slice-selection bias, the present study preferentially used multi-slice or volumetric parameters to provide a more comprehensive description of body composition distribution and to improve measurement stability and biological representativeness.

Recognizing that differences in scanner type, reconstruction parameters, and acquisition protocol may influence quantitative imaging phenotypes, we attempted to harmonize spatial resolution, intensity representation, and anatomic coverage during preprocessing, and subsequently screened abnormal outputs with rule-based checks followed by manual review when necessary. Although these steps cannot completely eliminate the technical variability inherent to real-world imaging data, they help improve the consistency, robustness, and transferability of body composition parameter extraction for downstream modeling.

#### 2.6.3 Definition of body composition parameters

Quantitative body composition features extracted from the automated segmentations included skeletal muscle area or volume, mean skeletal muscle attenuation, subcutaneous adipose tissue area or volume, visceral adipose tissue area or volume, and intermuscular adipose tissue area or volume. On this basis, additional derived indices reflecting structural relationships between tissue compartments, such as muscle-to-fat ratios, were further constructed. For cross-sectional measurements obtained from chest CT, thoracic vertebral levels with relative anatomic stability and good correspondence to conventional body composition methods were preferentially used. Previous studies have shown that middle-to-lower thoracic vertebral levels can to some extent substitute for the traditional abdominal L3 level for muscle-mass assessment while offering greater accessibility and consistency.

#### 2.6.4 Manual quality control of segmentation results and reproducibility assessment

To ensure that automated segmentation results were reliable and suitable for downstream analyses, a layered quality-control workflow was applied to all samples. First, all segmentation outputs underwent automated rule-based screening to identify abnormal anatomic coverage, values outside physiological plausibility ranges, or obviously distorted results. Samples failing automated screening were manually reviewed and retained or excluded according to predefined criteria.

Second, a randomly selected proportion of samples from both the development and validation cohorts underwent independent evaluation by two investigators experienced in thoracic imaging to assess the consistency of segmentation boundaries and quantitative outputs. Discrepant cases were resolved by discussion, and a senior radiologist adjudicated when necessary.

In a subset with available reference annotations, segmentation performance and measurement reproducibility were further quantified using metrics such as the Dice similarity coefficient, volumetric error, or intraclass correlation coefficient. The number of failed segmentations and their principal causes were also recorded and reported transparently.

### 2.7 Feature engineering and preprocessing of candidate predictors

Before model construction, all candidate predictors underwent unified preprocessing and feature engineering to reduce the impact of scale differences, skewed distributions, and variable redundancy on model fitting stability. Continuous variables were generally retained in their original form to preserve information and avoid the loss of statistical efficiency associated with arbitrary categorization. Potential non-linear relationships between continuous variables and the primary endpoint were assessed using restricted cubic splines. Functional transformation or segmented handling was applied only when there was clear evidence of non-linearity and a clinically interpretable rationale. Unless supported by a well-established clinical threshold, continuous variables were not arbitrarily dichotomized or converted into multiple categories.

Continuous variables with markedly skewed distributions were log-transformed or otherwise appropriately transformed according to their distributional characteristics to improve model stability. To facilitate joint modeling of variables measured on different scales and to reduce the influence of unit differences on parameter estimation, selected continuous variables were further standardized. For imaging-derived body composition parameters, indexation to height squared was considered where biologically meaningful and consistent with previous literature, so as to construct standardized indicators of relative body composition burden. In addition, to satisfy the requirement for scale consistency in multivariable modeling, relevant imaging quantitative features could also be standardized as z scores.

Because chest CT body composition analysis can generate multiple mutually correlated quantitative variables, body composition features were systematically evaluated for correlation and collinearity before model development. Highly correlated variables capturing similar biological dimensions were not entered into the same model simultaneously, in order to avoid information redundancy, parameter instability, and interpretive difficulty. Where several derived indicators reflected closely related source information, the priority of inclusion was prespecified on the basis of clinical interpretability, measurement stability, and biological representativeness, thereby controlling feature-dimensional inflation and reducing the risk of overfitting.

Through this preprocessing pipeline, we sought to improve the standardization, statistical suitability, and biological interpretability of candidate predictors while preserving the original information structure as much as possible, thereby providing a stable variable foundation for model development and validation.

### 2.8 Development of the nutritional risk prediction model

#### 2.8.1 Modeling strategy

The core analytical target of this study was a nutritional risk prediction model constructed from multidimensional candidate predictors. Candidate predictors included baseline clinical variables, nutritional indicators, immune-inflammatory indices, and AI-derived chest CT body composition parameters. The primary dependent variable for model construction was the 90-day nutrition-related adverse clinical trajectory. We developed candidate models across different informational layers to systematically evaluate the independent contribution and incremental value of automated chest CT body composition analysis in nutritional risk identification. GLIM-defined malnutrition was used descriptively for population characterization and was not incorporated as a model-building outcome.

Specifically, three levels of candidate models were developed: Model 1, a clinical-nutritional base model including routine clinical variables and basic nutritional indicators; Model 2, an imaging body composition model including only AI-derived CT body composition parameters; and Model 3, a multidimensional fusion model integrating AI body composition features on top of clinical, nutritional, and immune-inflammatory variables.

The purpose of this hierarchical strategy was to avoid the interpretive limitations of a single omnibus model and to allow clearer comparison of the contribution of different informational layers to nutritional risk identification. By sequentially constructing a base model, an imaging model, and a fusion model, we were able to directly assess whether AI-derived body composition information provided incremental discriminatory value beyond the conventional clinical and nutritional assessment framework. It should be further noted that the study distinguished methodologically between a “nutrition-anchored endpoint” and a “clinical application endpoint.” GLIM-defined malnutrition was mainly used as the nutritional construct anchor in model development to ensure interpretability and nutritional specificity, whereas clinical utility was primarily judged by the model's ability to identify short-term nutrition-related adverse clinical trajectories within 90 days. In other words, GLIM served as a methodological anchor rather than the sole clinically relevant target outcome. This strategy was chosen to balance the rigor of nutritional definitions with the practical needs of clinical risk identification, thereby enabling the model to reflect both nutritional vulnerability and the risk of near-term adverse clinical consequences.

#### 2.8.2 Variable selection and model fitting

Model development was conducted in the development cohort. Before modeling, the extent of missingness, distributional characteristics, and plausibility of all candidate predictors were systematically evaluated, and the candidate variable pool was predefined according to clinical interpretability, availability, and event-number constraints. Variable inclusion followed a combined principle of clinical knowledge and statistical restraint, so as to reduce the overfitting risk associated with purely data-driven selection.

Because the goal of this study was to build a nutritional risk stratification tool with strong clinical interpretability and transportability, multivariable logistic regression was used as the primary modeling approach. This method is appropriate for binary outcomes and also facilitates calculation of individualized risk probabilities and subsequent clinical translation. In settings with a relatively large number of candidate variables, strong inter-variable correlation, or potential overfitting risk, penalized regression was additionally used for shrinkage and selection to improve parameter stability and model generalizability.

During variable selection, predictors were not mechanically excluded solely on the basis of univariable results, nor was the outcome information overused. Variables with clear clinical relevance and prior evidence linking them to nutritional status or adverse outcomes were considered for retention even when their within-sample statistical association was relatively modest, with decisions informed by overall model performance. Through this approach, we aimed to preserve biological plausibility, statistical robustness, and clinical interpretability while maintaining model parsimony.

#### 2.8.3 Model output format

The final model was presented in a reproducible, interpretable, and clinically translatable format. For regression models, full coefficients, intercepts, and necessary calibration parameters were reported, allowing construction of an individualized risk-calculation formula. On this basis, the final model could also be visualized as a nomogram to improve readability and practical utility in clinical settings. Where feasible, an online calculator could further be developed to support rapid bedside or outpatient risk assessment. To ensure reproducibility, the model report also provides variable definitions, parameter expressions, and the method of risk estimation for future external validation and implementation.

### 2.9 Model evaluation and temporal validation

We systematically evaluated the predictive performance of each candidate model in the development cohort and then assessed it in an independent temporal validation cohort to examine stability, generalizability, and transportability. Model performance was assessed across multiple dimensions—discrimination, calibration, overall prediction error, and clinical utility—rather than relying on a single metric.

Discrimination was quantified by the area under the receiver operating characteristic curve (AUC). Calibration was assessed using calibration plots, intercept, and slope. Overall performance was evaluated using the Brier score. Decision curve analysis was performed to compare net benefit across threshold probabilities and evaluate clinical utility.

To assess the added value of AI-derived body composition information beyond conventional nutritional assessment, we compared the performance of Models 1, 2, and 3 in both cohorts, focusing on differences in discrimination, calibration, and net benefit. Net reclassification improvement (NRI) and integrated discrimination improvement (IDI) were also calculated where appropriate but were not used as the sole basis for claiming superiority.

A strict temporal validation strategy was adopted. All model-building steps—candidate predictor selection, parameter estimation, and final model specification—were performed exclusively in the development cohort. The temporal validation cohort was used only for performance evaluation and was not involved in any model retraining or reselection, reducing optimism bias associated with random splitting. Internal correction using resampling-based methods was performed to estimate overfitting and obtain stable performance metrics.

### 2.10 Prognostic bridging analysis

To evaluate the longitudinal clinical validity of the nutritional risk model, a prognostic bridging analysis was performed to determine whether model-derived risk strata could identify subgroups with poorer long-term outcomes. Individualized risk scores were calculated using the parameters from model development, and patients were categorized into risk strata according to prespecified thresholds.

Kaplan–Meier curves were generated for overall survival (OS) and progression-free survival (PFS), and survival differences were compared using the log-rank test. Cox proportional hazards regression was used to evaluate the association between risk score and strata with OS and PFS, reporting hazard ratios and 95% confidence intervals. Multivariable Cox models adjusted for key clinical covariates to assess the independent contribution of nutritional risk stratification. The proportional hazards assumption was examined using Schoenfeld residuals.

The prognostic bridging analysis aimed to test whether the nutritional risk framework reflected both short-term adverse clinical trajectories and broader long-term prognostic risk, thereby extending clinical validity without altering the role of the nutritional risk model as the primary model.

### 2.11 Missing data, bias control, and sensitivity analyses

The extent and pattern of missingness were systematically described, and the plausibility of a random missing-data mechanism was assessed. For key variables with low missingness meeting imputation assumptions, multiple imputation was performed to reduce sample loss and estimation bias. Main analyses were repeated in the complete-case dataset as a sensitivity check.

Several design-stage measures were prespecified to minimize selection and information bias, including uniform baseline assessment windows, endpoint adjudication criteria, laboratory collection windows, and imaging quality-control standards. A strict temporal split was used for model development and validation to reduce optimism bias.

Sensitivity analyses were performed to test robustness. First, high nutritional risk defined by NRS-2002 was used as an alternative outcome. Second, analyses were repeated within subgroups receiving the same initial treatment pathway. Third, models based on two-dimensional vs. three-dimensional body composition parameters were compared. Fourth, analyses were repeated in subgroups defined by tumor stage, pathological subtype, and sex. To specifically address the heterogeneity of the primary composite endpoint, we performed additional sensitivity analyses using each individual component of the composite endpoint as the alternative outcome. Specifically, we refitted the three models (clinical-nutritional, imaging, and fusion) using: (1) treatment delay/dose reduction/interruption alone; (2) unplanned hospitalization alone; (3) grade ≥3 adverse events alone; and (4) 90-day mortality alone as the binary outcome. Given the limited event numbers for individual components, we also performed a leave-one-component-out analysis, in which the composite endpoint was redefined excluding each component in turn, to evaluate whether any single component disproportionately drove the model's performance.

Supplementary sensitivity analyses using alternative supervised learning methods (e.g., tree-based ensemble algorithms) were also performed and compared against the main multivariable logistic regression model to determine whether the main predictors and model discrimination remained directionally consistent across different modeling frameworks.

Through missing-data handling, bias control, and multilevel sensitivity analyses, we sought to evaluate the dependence of core findings on analytical assumptions, variable definitions, and patient compositions, thereby improving the robustness, transparency, and interpretability of the study conclusions.

To further exclude the possibility that our findings were influenced by the choice of outcome definition, we conducted a sensitivity analysis using GLIM-defined malnutrition as the dependent variable in the same modeling framework. This analysis was exploratory and intended to benchmark our model's performance against an established nutritional diagnostic standard.

### 2.12 Statistical analysis

All statistical analyses were conducted according to a prespecified analytical plan. Continuous variables were summarized according to their distributional characteristics, using mean ± standard deviation for normally distributed data and median with interquartile range for non-normally distributed data; categorical variables were summarized as counts and percentages. Group comparisons were performed using methods appropriate to variable type and distribution, including the independent-samples *t* test or Wilcoxon rank-sum test for continuous variables, and the chi-square test or Fisher's exact test for categorical variables. All statistical tests were two-sided, and *P* < 0.05 was considered statistically significant.

Model-related analyses followed reporting principles for prediction-model research. In addition to significance testing, effect sizes and 95% confidence intervals were emphasized to enhance interpretability and comparability. Model performance was reported according to the predefined evaluation framework, including metrics related to discrimination, calibration, and clinical utility, and results were presented separately for the development and temporal validation cohorts.

All analyses were performed in R software (R Foundation for Statistical Computing, Vienna, Austria) using validated statistical packages for data processing, model development, and performance evaluation. The analytical workflow was prespecified before study implementation to reduce bias related to data-driven analyses and improve reproducibility.

## 3 Results

### 3.1 Study population screening and cohort composition

A total of 1,128 patients with pathologically confirmed lung cancer were identified. After individual screening, 472 were excluded for not meeting the study criteria. The main reasons for exclusion were baseline CT outside the required time window (*n* = 154), missing laboratory data (*n* = 83), missing height, weight, or nutrition-screening information (*n* = 71), inadequate image quality (*n* = 49), pathological type not meeting the inclusion criteria (*n* = 62), concomitant active primary malignancy other than NSCLC (*n* = 15), obvious anatomic or technical factors affecting body composition quantification (*n* = 10), and absence of follow-up data sufficient for endpoint adjudication (*n* = 28). Ultimately, 656 patients with NSCLC were included, of whom 432 comprised the development cohort and 224 the independent temporal validation cohort (Figure 1).

Baseline characteristics were well balanced between the development and validation cohorts. The median age was 64 years (57–71), with 62.0% male, and no significant differences were observed between cohorts (*P* = 0.312 and *P* = 0.842, respectively). Lung adenocarcinoma was the most common pathological subtype (58.4%), followed by squamous cell carcinoma (33.8%), with similar distributions across cohorts (*P* = 0.764). Stage III–IV disease accounted for 64.3% of the overall population (63.9% in the development cohort vs. 65.2% in the validation cohort, *P* = 0.781), indicating a predominantly intermediate-to-advanced disease population. ECOG PS ≥2 was present in 28.5% of patients, with no inter-cohort difference (*P* = 0.538). Treatment approaches were heterogeneous but similarly distributed between cohorts (*P* = 0.608). Overall, no significant inter-cohort differences were observed in demographic, tumor, or treatment characteristics, suggesting that the temporal split did not introduce major systematic bias and provided a stable basis for model development (Table 1).

**Table 1:** Baseline characteristics and key endpoint distribution of the study population.

Variable	Overall (*n* = 656)	Development (*n* = 432)	Validation (*n* = 224)	*P* value
Clinical characteristics				
Age, years	64 (57–71)	64 (57–70)	65 (58–72)	0.312
Male, *n* (%)	407 (62.0)	266 (61.6)	141 (62.9)	0.842
Smoking history, *n* (%)	359 (54.7)	235 (54.4)	124 (55.4)	0.693
ECOG PS ≥ 2, *n* (%)	187 (28.5)	120 (27.8)	67 (29.9)	0.538
Comorbidities ≥ 2, *n* (%)	206 (31.4)	134 (31.0)	72 (32.1)	0.721
Tumor characteristics				
Adenocarcinoma, *n* (%)	383 (58.4)	250 (57.9)	133 (59.4)	0.764
Squamous carcinoma, *n* (%)	222 (33.8)	149 (34.5)	73 (32.6)	
Others, *n* (%)	51 (7.8)	33 (7.6)	18 (8.0)	
Stage I–II, *n* (%)	234 (35.7)	156 (36.1)	78 (34.8)	0.781
Stage III–IV, *n* (%)	422 (64.3)	276 (63.9)	146 (65.2)	
Treatment				
Surgery, *n* (%)	185 (28.2)	124 (28.7)	61 (27.2)	0.608
Chemotherapy, *n* (%)	161 (24.5)	105 (24.3)	56 (25.0)	
Radiotherapy/CCRT, *n* (%)	124 (18.9)	83 (19.2)	41 (18.3)	
Targeted therapy, *n* (%)	107 (16.3)	70 (16.2)	37 (16.5)	
Immunotherapy, *n* (%)	79 (12.1)	50 (11.6)	29 (13.0)	
Nutritional variables				
BMI, kg/m^2^	22.3 (20.1–24.8)	22.4 (20.2–24.7)	22.2 (20.0–24.9)	0.658
Weight loss, %	6.8 (3.2–11.5)	6.7 (3.1–11.3)	7.1 (3.4–11.8)	0.447
Albumin, g/L	37.2 ± 5.1	37.3 ± 5.0	37.0 ± 5.3	0.512
PNI	45.1 ± 6.8	45.3 ± 6.6	44.8 ± 7.1	0.429
NRS-2002 ≥ 3, *n* (%)	257 (39.2)	171 (39.6)	86 (38.4)	0.768
GLIM malnutrition, *n* (%)	238 (36.3)	158 (36.6)	80 (35.7)	0.812
Key endpoint				
Short-term nutrition-related adverse clinical trajectory, *n* (%)	221 (33.7)	148 (34.3)	73 (32.6)	0.637
Inflammatory markers				
NLR	3.6 (2.2–5.4)	3.5 (2.1–5.3)	3.7 (2.3–5.6)	0.334
SII	875 (560–1,320)	860 (550–1,295)	910 (580–1,360)	0.298
Imaging parameters				
SMI, cm^2^/m^2^	42.6 ± 8.7	42.8 ± 8.6	42.3 ± 8.9	0.541
Muscle attenuation, HU	36.5 ± 7.2	36.7 ± 7.1	36.2 ± 7.4	0.489

### 3.2 Baseline characteristics and distribution of endpoint events

In the development cohort, 158 patients (36.6%) were classified as malnourished by GLIM criteria, and 171 (39.6%) were at high nutritional risk (NRS-2002 ≥ 3). In the validation cohort, these rates were 35.7% and 38.4%, respectively, with no significant differences between cohorts (*P* = 0.812 and *P* = 0.768) (Table 1). The primary endpoint—short-term nutrition-related adverse clinical trajectory—occurred in 148 patients (34.3%) in the development cohort and 73 (32.6%) in the validation cohort (*P* = 0.637) (Table 1). Among composite endpoint components, treatment delay, dose reduction, or unplanned interruption was most common, followed by unplanned hospitalization and grade ≥ 3 adverse events (Supplementary Table S1).

Among the 148 patients in the development cohort who experienced the primary composite endpoint, the distribution of individual components was as follows: treatment delay, dose reduction, or unplanned interruption occurred in 92 patients (62.2%); unplanned hospitalization in 51 patients (34.5%); postoperative complications of Clavien–Dindo grade ≥ II in 19 patients (12.8%); grade ≥ 3 treatment-related adverse events in 42 patients (28.4%); and all-cause death within 90 days in 13 patients (8.8%). Notably, 47 patients (31.8%) experienced more than one component event during the 90-day follow-up period. In the temporal validation cohort, the component distribution was similar: treatment interruption in 44 patients (60.3%), unplanned hospitalization in 24 (32.9%), postoperative complications in 10 (13.7%), grade ≥3 adverse events in 20 (27.4%), and 90-day mortality in 6 (8.2%).

Stratified analysis showed that patients with GLIM-defined malnutrition had significantly worse clinical, nutritional, inflammatory, and body composition profiles. They were older, had poorer ECOG PS, and higher rates of advanced-stage disease (all *P* < 0.01); greater weight loss and lower albumin and PNI levels (all *P* < 0.001); higher NLR and SII values (both *P* < 0.001); and lower skeletal muscle index and muscle attenuation but higher intermuscular adipose tissue volume and visceral adiposity index (all *P* < 0.01), indicating that malnutrition is closely linked to inflammatory activation and adverse body composition remodeling (Supplementary Table S1).

Of note, GLIM-defined malnutrition was used for descriptive purposes only and was not employed as the outcome for model development. The primary model-building outcome was the 90-day composite endpoint. This analytical separation was intentionally designed to prevent label leakage that could arise from using imaging-derived body composition parameters to both define and predict malnutrition status, thereby ensuring a more rigorous evaluation of the incremental predictive value of AI-derived body composition features.

### 3.3 Quantification of nutrition-related body composition phenotypes from chest CT and assessment of measurement reliability

All CT images underwent standardized preprocessing and AI-driven body composition quantification. Automated screening identified 17 cases (2.6%) with potentially abnormal outputs; 6 were excluded due to segmentation distortion or implausible values, and 11 were accepted after manual review. Complete body composition parameters were obtained for 650 patients (99.1% success rate).

In the quality-control subset, automated quantification showed good agreement with manual assessment. Median Dice similarity coefficients were 0.94 (0.91–0.96) for skeletal muscle, 0.96 (0.94–0.97) for subcutaneous fat, and 0.95 (0.92–0.97) for visceral fat (Supplementary Figure S1A), with no significant differences between cohorts (Supplementary Figure S1B). Intraclass correlation coefficients ranged from 0.93 to 0.95, all exceeding 0.90 (Supplementary Figure S1C).

### 3.4 Preprocessing of candidate nutrition-related phenotypes and feature-selection results

For inflammation-related indicators with skewed distributions, CRP, NLR, PLR, and SII were log-transformed. Restricted cubic spline analyses showed generally stable relationships with the primary endpoint: log(CRP) was mildly non-linear, log(NLR) borderline non-linear, log(PLR) linear, and log(SII) somewhat more non-linear (Supplementary Figures S2A–D), with no clear threshold effects, supporting their inclusion as continuous phenotypes in modeling.

Correlation analysis showed skeletal muscle volume and index were highly correlated (r > 0.75), as were visceral adipose area and index, with variance inflation factor values exceeding 5 for some variables (Supplementary Table S2). To reduce redundancy and improve model stability, skeletal muscle index, skeletal muscle attenuation, intermuscular adipose tissue volume, and visceral adiposity index were retained for modeling, reflecting muscle reserve, muscle quality, ectopic fat infiltration, and fat distribution, respectively. Variable selection combined clinical prespecification and penalized shrinkage rather than single-step significance screening (Supplementary Table S2).

### 3.5 Development of the nutritional risk stratification model

In the development cohort, 148 of 432 patients (34.3%) experienced the primary endpoint within 90 days. Based on the prespecified variable framework, three risk-stratification models were built: a clinical-nutritional model, an imaging body composition model, and a fusion model. These models represented, respectively, the conventional nutritional assessment dimension, the imaging nutritional phenotype dimension, and the integrated nutritional-inflammatory-body composition dimension, and were developed to compare the capacity of different informational layers to identify short-term nutrition-related adverse clinical trajectories (Table 2).

**Table 2:** Multivariable logistic regression models for prediction of 90-day adverse clinical trajectory.

Variable	Model 1 OR (95% CI)	*P*	Model 2 OR (95% CI)	*P*	Model 3 OR (95% CI)	*P*
Age (per year)	1.02 (1.00–1.04)	0.042			1.01 (0.99–1.03)	0.218
ECOG PS ≥2	1.76 (1.12–2.76)	0.015			1.41 (0.88–2.26)	0.153
Weight loss (per 5%)	1.28 (1.12–1.46)	< 0.001			1.24 (1.08–1.42)	0.002
BMI	0.96 (0.92–1.00)	0.061			0.98 (0.93–1.03)	0.412
PNI	0.94 (0.91–0.97)	< 0.001			0.95 (0.92–0.98)	0.003
log(SII)	1.21 (1.08–1.36)	0.001			1.18 (1.05–1.33)	0.006
Skeletal muscle index			0.89 (0.84–0.94)	< 0.001	0.91 (0.86–0.96)	0.001
Muscle attenuation			0.92 (0.87–0.97)	0.003	0.94 (0.89–0.99)	0.021
Intermuscular fat volume			1.17 (1.10–1.24)	< 0.001	1.14 (1.07–1.22)	< 0.001
Visceral fat index			1.10 (1.04–1.16)	0.002	1.08 (1.02–1.15)	0.011

OR, odds ratio; CI, confidence interval; ECOG PS, Eastern Cooperative Oncology Group performance status; BMI, body mass index; PNI, prognostic nutritional index; SII, systemic immune-inflammation index.

In the clinical-nutritional model, percentage weight loss (OR 1.28 per 5%, *P* < 0.001), PNI (OR 0.94, *P* < 0.001), and log(SII) (OR 1.21, *P* = 0.001) were independent predictors (Table 2). In the imaging body composition model, skeletal muscle index (OR 0.89, *P* < 0.001), skeletal muscle attenuation (OR 0.92, *P* = 0.003), intermuscular adipose tissue volume (OR 1.17, *P* < 0.001), and visceral adiposity index (OR 1.10, *P* = 0.002) were all significant (Table 2). In the fusion model, percentage weight loss (OR 1.24, *P* = 0.002), PNI (OR 0.95, *P* = 0.003), log(SII) (OR 1.18, *P* = 0.006), skeletal muscle index (OR 0.91, *P* = 0.001), and intermuscular adipose tissue volume (OR 1.14, *P* < 0.001) retained independent predictive effects, while BMI was no longer significant (*P* = 0.412) (Table 2).

**Figure 2:**
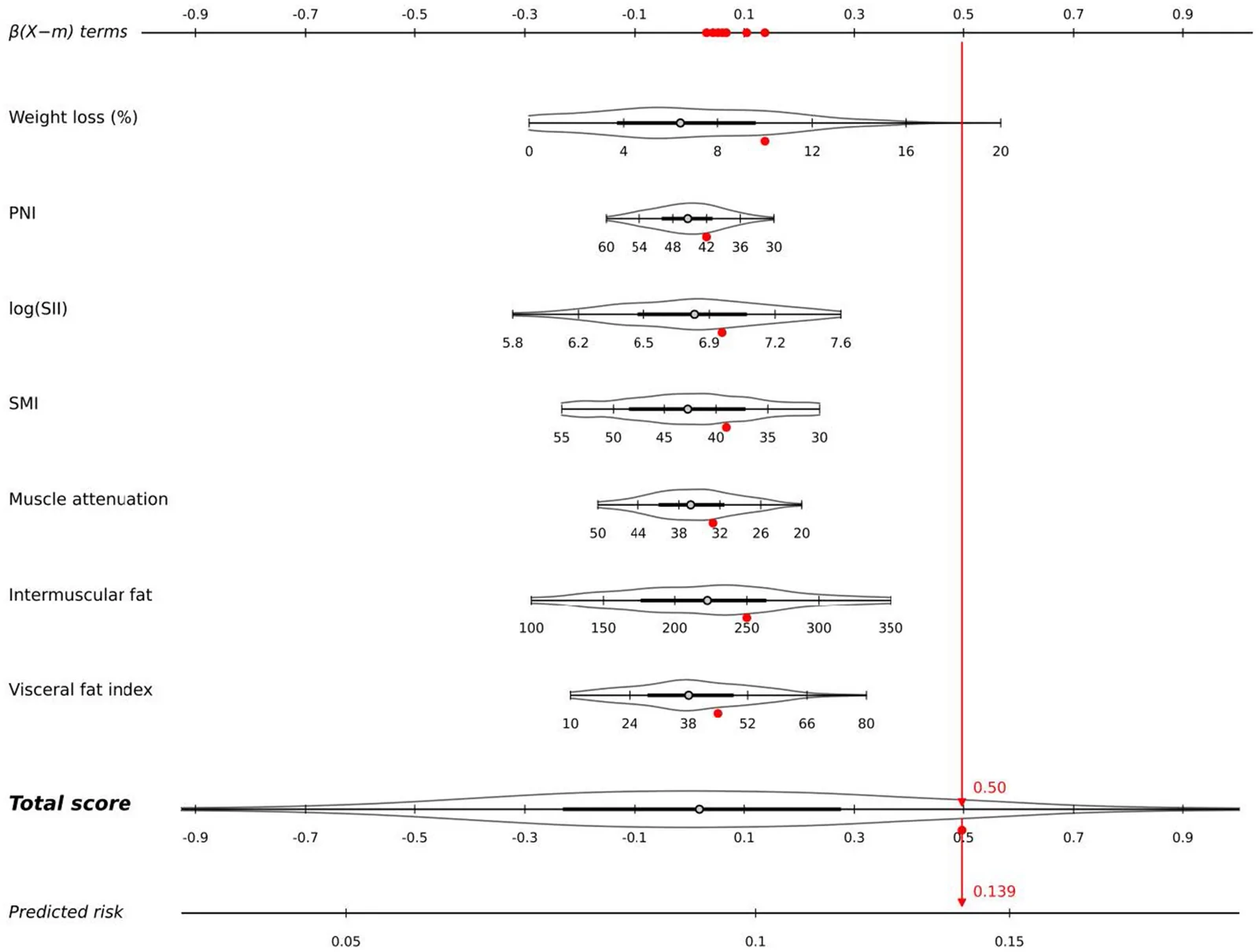
Prediction of nutritional risk based on the integrated model.

The final model coefficients and intercept are shown in Table 3, with an individualized risk prediction formula. A nomogram was constructed to visualize variable contributions (Figure 2). Percentage weight loss and intermuscular adipose tissue volume had the broadest contribution ranges, indicating greater weight in risk stratification, while PNI and skeletal muscle attenuation contributed directionally consistent effects. The relationship between total score and predicted risk was continuously non-linear, with steeper risk increases at higher scores (Figure 2).

**Table 3:** Final integrated model coefficients and risk equation.

Variable	β coefficient	OR (95% CI)	*P* value	Assigned points^*^
Intercept	−2.318			
Weight loss (per 5%)	0.215	1.24 (1.08–1.42)	0.002	24
PNI (per 1 unit)	−0.051	0.95 (0.92–0.98)	0.003	18
log(SII)	0.166	1.18 (1.05–1.33)	0.006	19
Skeletal muscle index	−0.094	0.91 (0.86–0.96)	0.001	21
Muscle attenuation	−0.062	0.94 (0.89–0.99)	0.021	14
Intermuscular fat volume	0.131	1.14 (1.07–1.22)	< 0.001	27
Visceral fat index	0.077	1.08 (1.02–1.15)	0.011	16

Risk equation: logit(P) = −2.318 + 0.215 × [weight loss per 5%] – 0.051 × [PNI] + 0.166 × [log(SII)] – 0.094 × [skeletal muscle index] – 0.062 × [muscle attenuation] + 0.131 × [intermuscular fat volume] + 0.077 × [visceral fat index]. Predicted probability: P = exp(logit) / (1 + exp(logit)). ^*^Assigned points were scaled to a 0–100 nomogram framework according to the relative magnitude of the β coefficients.

### 3.6 Evaluation of nutritional risk identification and temporal validation

**Figure 3:**
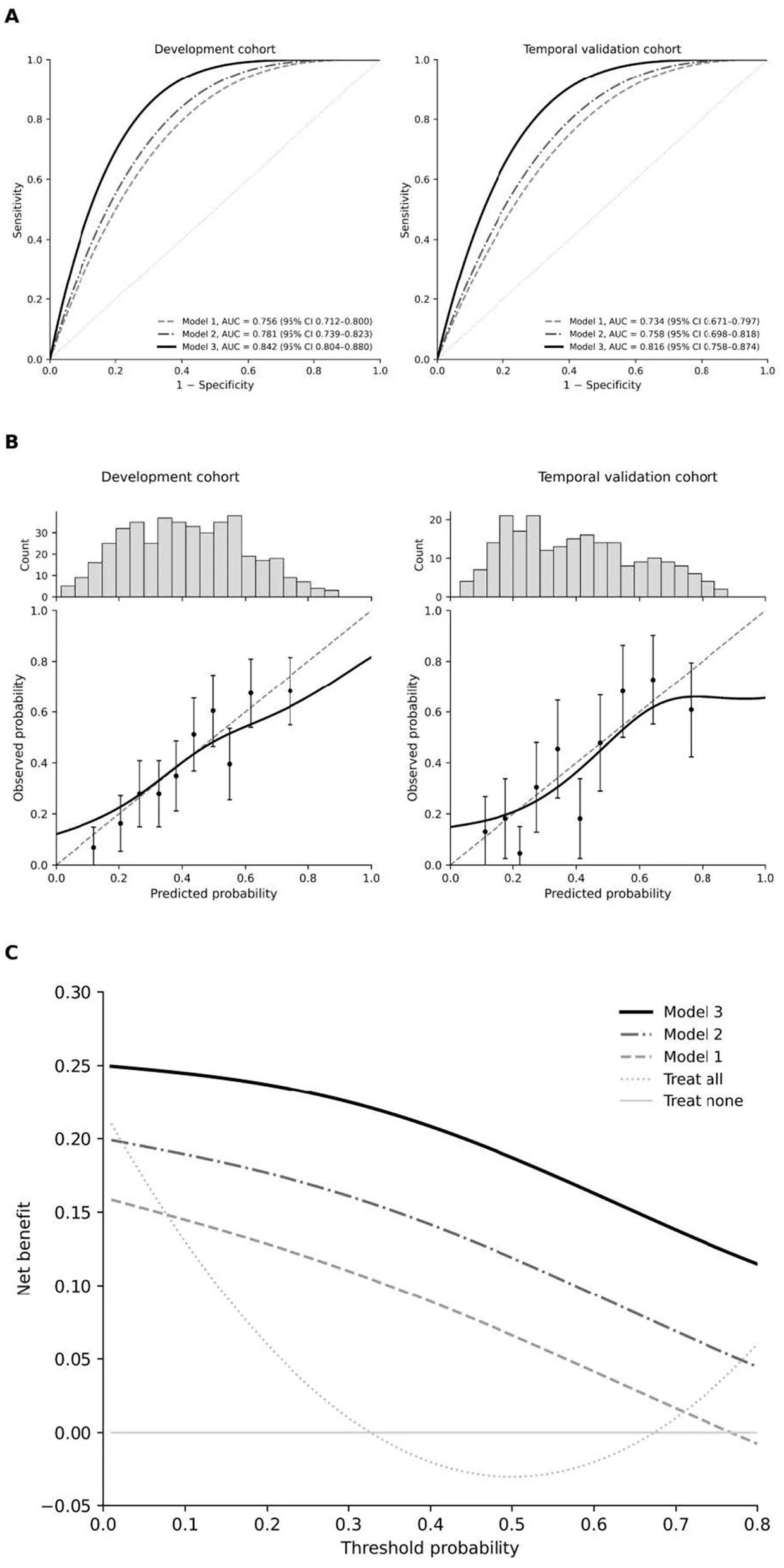
Evaluation of model discrimination, calibration, and clinical decision utility. **(A)** Receiver operating characteristic curves were used to assess the discriminative performance of the three models in the development cohort and the temporal validation cohort. **(B)** Calibration curves were used to evaluate the agreement between predicted probabilities and observed event rates. **(C)** Decision curve analysis was used to assess the clinical net benefit of different models across a range of threshold probabilities.

In the development cohort, the AUCs for Models 1, 2, and 3 were 0.756, 0.781, and 0.842, respectively; in the temporal validation cohort, the corresponding AUCs were 0.734, 0.758, and 0.816 (Figure 3A). DeLong testing showed the fusion model significantly outperformed the other two models in both cohorts (ΔAUC 0.086 and 0.061 in development; 0.082 and 0.058 in validation; all *P* < 0.01).

Calibration analysis showed the fusion model had a calibration slope of 0.97 and intercept of 0.02 in the development cohort, and 0.94 and 0.04 in the validation cohort, indicating good agreement between predicted and observed risks (Figure 3B). Brier scores for the fusion model were 0.167 and 0.173, lower than those for Models 1 and 2, reflecting more stable probability estimation (Figure 3B). Decision curve analysis showed the fusion model achieved the highest net benefit across threshold probabilities from 0.10 to 0.60, with advantages most pronounced in the moderate-to-high threshold range (Figure 3C).

In the temporal validation cohort, the fusion model also showed significant improvement in risk reclassification relative to the clinical-nutritional model. The net reclassification improvement was 0.186 (*P* = 0.004), and the integrated discrimination improvement was 0.072 (*P* = 0.008), indicating that the introduction of AI-derived body composition information significantly enhanced nutritional risk stratification (Supplementary Table S3). Further analysis showed that the fusion model more often reassigned event cases upward in risk while reducing unnecessary high-risk classification among non-event cases, thereby improving both the accuracy and the clinical applicability of nutritional risk identification (Supplementary Table S3).

### 3.7 Prognostic bridging analysis of nutritional risk phenotypes

**Figure 4:**
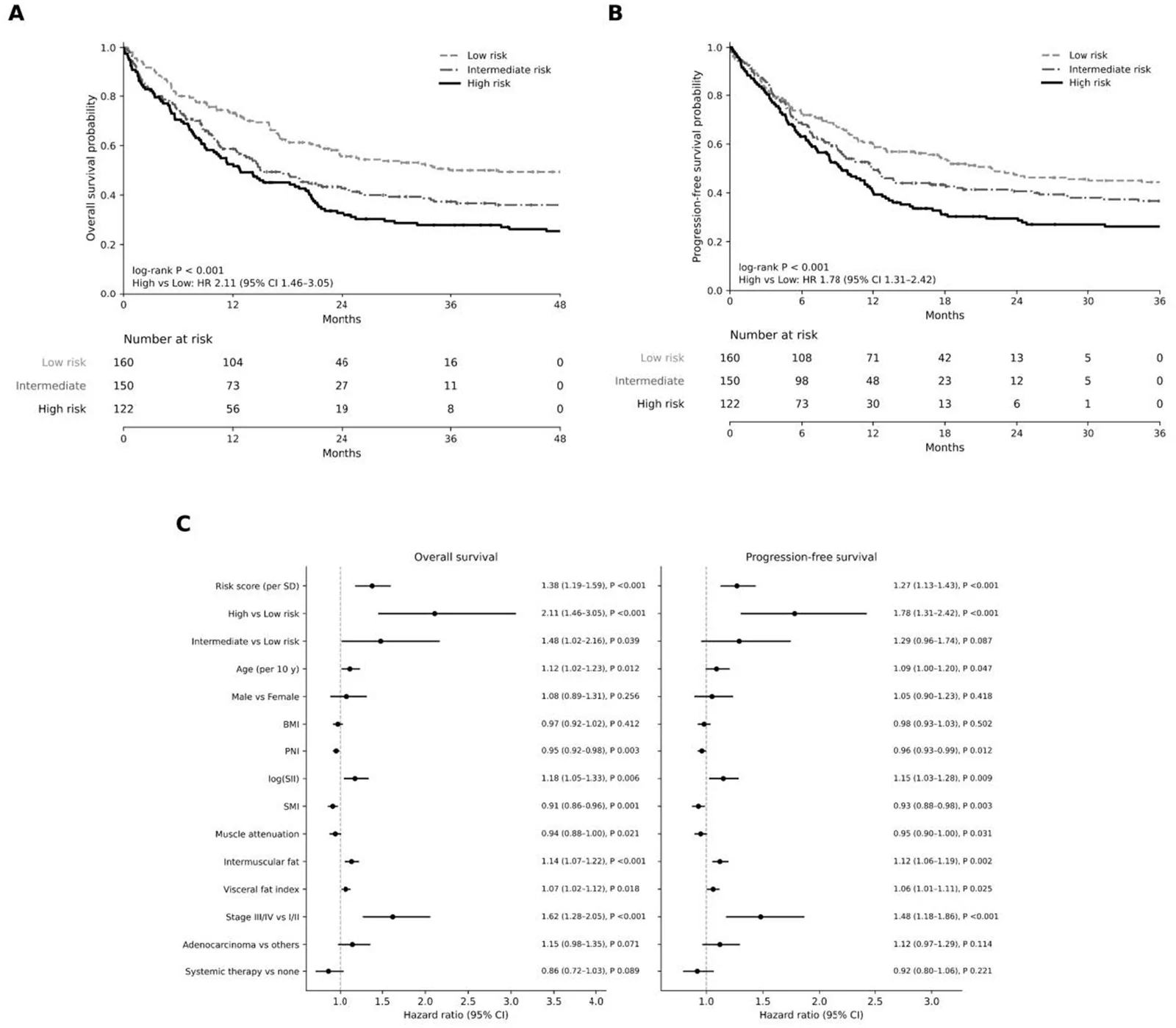
Survival analysis and multivariable Cox regression results based on nutritional risk phenotypes. **(A)** Overall survival curves for patients stratified by nutritional risk. **(B)** Progression-free survival curves for patients stratified by nutritional risk. **(C)** Forest plot of multivariable Cox regression analysis showing the associations of the nutritional risk score and related covariates with overall survival and progression-free survival.

Using the fusion model, patients were categorized into low-, intermediate-, and high-risk groups. Kaplan–Meier analysis showed significantly shorter overall survival in the high-risk group (median 18.6 months) compared with the intermediate- (31.4 months) and low-risk groups (not reached) (log-rank *P* < 0.001) (Figure 4A). A similar pattern was observed for progression-free survival (median 9.7, 15.8, and 24.6 months, respectively; *P* < 0.001) (Figure 4B).

In Cox regression, each 1-SD increase in risk score was associated with a 38% higher risk of death (HR 1.38, *P* < 0.001) and a 27% higher risk of progression or death (HR 1.27, *P* < 0.001) (Figure 4C). The risk score remained an independent predictor after adjusting for age, sex, BMI, PNI, log(SII), body composition parameters, and clinical stage. Compared with the low-risk group, the high-risk group had an adjusted HR of 2.11 for all-cause death (*P* < 0.001) and 1.78 for progression or death (*P* < 0.001); the intermediate-risk group showed an HR of 1.48 for death (*P* = 0.039) but a non-significant HR of 1.29 for progression (*P* = 0.087) (Figure 4C). Covariates showed directionally consistent associations, with intermuscular adipose tissue volume and log(SII) linked to worse prognosis, while skeletal muscle index and PNI were protective (Figure 4C).

### 3.8 Robustness of nutritional risk assessment, bias control, and sensitivity analyses

Missing data rates were low (< 5% for all major indicators). After multiple imputation, parameter estimates and predictive performance remained consistent with complete-case analysis; in the fusion model, percentage weight loss (OR 1.23) and PNI (OR 0.95) were essentially unchanged (Supplementary Table S4).

Sensitivity analyses confirmed model robustness. Using NRS-2002 as an alternative endpoint, the fusion model achieved AUCs of 0.829 and 0.801 in the development and validation cohorts, respectively (Supplementary Table S4). When restricted to patients receiving standardized treatment pathways, performance remained stable (AUC 0.836, calibration slope 0.96, Brier score 0.169). Replacing two-dimensional indices with three-dimensional volumetric parameters slightly improved discrimination (AUC 0.854), with no change in the direction or significance of main predictors (Supplementary Table S4). Across sensitivity analyses, skeletal muscle index and PNI remained protective, while intermuscular adipose tissue volume and inflammatory indices remained associated with increased risk.

Subgroup analyses showed stable model performance across strata defined by age, sex, tumor stage, treatment modality, and baseline nutritional status, with AUCs ranging from 0.802 to 0.861 and no significant heterogeneity (all interaction *P* > 0.10), indicating good generalizability (Supplementary Table S5).

Sensitivity analyses using individual components of the composite endpoint as alternative outcomes showed that the fusion model maintained acceptable discriminative performance across all event types: AUCs ranged from 0.789 (for 90-day mortality) to 0.836 (for treatment interruption), with overlapping confidence intervals indicating no single component dominated the model's predictive signal. Leave-one-component-out analyses yielded AUCs ranging from 0.827 to 0.840 in the development cohort and 0.802 to 0.814 in the validation cohort, all within the 95% CI of the primary analysis (AUC 0.842, 95% CI 0.807–0.877), suggesting that the model's performance was not driven disproportionately by any single component of the composite endpoint.

## 4 Discussion

This study focused on nutritional vulnerability and outcome heterogeneity in patients with NSCLC, a host-related problem that has long been underestimated despite being highly amenable to intervention. Conventional nutritional screening and stratification rely largely on weight change, BMI, and a limited number of laboratory indicators, and are therefore unable to identify high-risk individuals whose body weight appears relatively stable despite substantial remodeling of muscle and adipose tissue. Using routine chest CT for opportunistic extraction of body composition phenotypes, combined with peripheral blood immune-inflammatory and nutritional indices, we developed and validated a multidimensional nutritional risk model for risk stratification. The results show that the fusion model identified patients more likely to experience short-term nutrition-related adverse clinical trajectories as well as poor long-term prognosis, and that its performance remained stable in temporal validation. These findings support AI-driven CT body composition modeling as an important methodological complement to oncologic nutritional assessment and provide reproducible evidence for more proactive and individualized supportive care.

The central finding of the present study is that a multimodal fusion model integrating chest CT body composition phenotypes, conventional nutritional assessment indicators, and immune-inflammatory signals enabled refined stratification of both short-term nutrition-related adverse clinical trajectories and long-term survival outcomes before treatment or early during treatment, and that this performance remained consistent across multiple model comparisons and sensitivity analyses. This suggests that one-dimensional nutritional or inflammatory assessment alone is insufficient to capture the complex host state of patients with NSCLC, whereas body composition, as a key intermediary linking metabolic reserve and immune function, can substantially enhance risk identification within a multimodal framework. Mechanistically, muscle mass and fat distribution not only reflect energy reserve, but are also deeply involved in inflammatory regulation and immune response, making their joint modeling with systemic inflammatory markers biologically plausible. Compared with prior work, the present study further emphasizes the predictive value of body composition information in the temporal dimension, namely its ability to capture the risk of adverse outcomes before overt progression rather than merely serving as a late-stage consequence marker. Recent studies have shown that body composition phenotypes derived from automated CT analysis can predict pathologic response after neoadjuvant immunotherapy, suggesting that they may serve not only as perioperative risk markers but also as tools for stratifying benefit from immunotherapy (28). Additional multicenter studies have reported that deep-learning-driven sarcopenia assessment is associated with severe treatment toxicity and poor survival and has been validated in external datasets, indicating good cross-population generalizability of automated body composition analysis (29). Likewise, large dual-center studies have shown that body composition indices retain stable prognostic value across disease stages and centers and in some settings outperform traditional single-slice measurements, methodologically supporting the need for three-dimensional or multi-region modeling (30). Larger pan-cancer analyses have further confirmed that fully automated body composition indicators retain independent predictive value in external validation, underscoring their potential for integration into standard oncologic workflows (31). Building on this foundation, the present study incorporated immune-inflammatory indicators and an AI-based fusion modeling strategy to move from a single imaging phenotype toward a multidimensional host representation, thereby improving predictive accuracy while also shedding light on interactions among nutritional status, body composition, and the immune microenvironment. In this way, the model may provide a more interpretable quantitative tool for precision nutritional intervention and individualized treatment decision-making.

From a biological and nutritional perspective, the improved stratification achieved by our model appears to derive from simultaneous integration of skeletal muscle quantity, skeletal muscle quality, and adipose phenotypes, rather than from simple accumulation of isolated body composition indices. Traditional nutritional assessment tends to emphasize outward manifestations such as body weight or BMI, but our results suggest that the key determinants of host vulnerability may lie in the metabolically dysregulated state jointly reflected by depletion of muscle reserve, fatty infiltration of muscle, and abnormal remodeling of adipose tissue. Previous studies have shown that in advanced NSCLC, patients with both high intermuscular fat infiltration and low muscle density have the worst overall survival, indicating that the abnormal information represented by impaired muscle quality cannot be replaced by muscle area alone (32). This is consistent with our finding that skeletal muscle index and intermuscular adipose tissue volume both entered the final model and retained independent effects, suggesting that nutritional risk is not simply a manifestation of sarcopenia but rather a composite imbalance phenotype marked by coexisting muscle depletion and ectopic fat deposition. Similarly, in resectable NSCLC, markers of myosteatosis extracted from preoperative chest CT have been shown to be independently associated with postoperative survival and to improve prognostic assessment (33), underscoring the clinical accessibility and interpretive value of body composition information embedded in routine chest CT. The significance of adipose tissue in our study likewise should not be understood merely in terms of energy storage, but rather as a composite reflection of inflammation-driven lipid mobilization, tissue remodeling, and metabolic adaptability. Prior studies have reported that preoperative adipopenia is independently associated with poor prognosis in early-stage pathological NSCLC (34), and when imaging body composition is jointly modeled with inflammatory and nutritional indices, prediction of early postoperative recurrence is improved beyond single-domain indicators (35). In patients receiving systemic therapy, CT-derived fat and muscle parameters provide more discriminative prognostic information than BMI (36), and increased intermuscular fat is associated with higher mortality, reduced treatment accessibility, and greater symptom burden, whereas subcutaneous fat shows a protective association in the opposite direction (37). Taken together, these findings suggest that the biological significance of different adipose compartments and their qualitative changes is not the superficial distinction between obesity and leanness, but whether the host still possesses the metabolic resilience needed to withstand tumor burden and treatment stress. The superiority of our fusion model over either the clinical-nutritional or imaging-only model indicates that the clinical meaning of body composition abnormalities can be more fully recognized only when interpreted in the interactive context of nutrition and inflammation. Further studies have shown that AI-segmented body composition parameters can be linked in an interpretable manner to circulating inflammatory signals and molecular features, offering biological clues as to how body composition may influence clinical outcomes through immunometabolic pathways (38). Thus, what our study reveals is not a localized effect of any single muscle or fat indicator, but a more physiologically faithful overall characterization of nutritional reserve, metabolic remodeling, and host vulnerability in NSCLC centered on opportunistic chest CT phenotyping.

The inclusion of immune-inflammatory indicators in the nutritional risk stratification framework is meaningful not merely because it adds several peripheral blood variables, but because it further clarifies that nutritional risk in NSCLC is essentially a host-vulnerability state jointly shaped by body composition abnormalities, inflammatory activation, and immune disequilibrium. Prior evidence shows that in NSCLC treated with immune checkpoint inhibitors, cachexia is significantly associated with poor prognosis and that this association is independent of tumor PD-L1 expression, suggesting that a key determinant of treatment outcome may lie not only in the tumor immune phenotype itself but also in the host nutritional-inflammatory background (39). Additional studies have shown that multiple cachexia-related inflammatory and metabolic factors are stably associated with immunotherapy outcomes, supporting the view that inflammatory-nutritional signals are biologically grounded predictive information rather than merely accompanying abnormalities (40). When nutritional status, inflammatory status, and muscle-mass change are considered within the same analytical framework, their relationship with immunotherapy benefit and survival outcomes becomes clearer, indicating that nutritional vulnerability is not a linear extrapolation of any single abnormal indicator, but rather a composite phenotype of multisystem imbalance (41). This understanding is highly consistent with our results, wherein the fusion model outperformed one-dimensional models, suggesting that immune-inflammatory information provides an essential biological interpretive layer for body composition phenotypes. Sarcopenia and related body composition abnormalities have already been shown to be associated with poorer survival after immunotherapy (42), and ongoing muscle loss during treatment may further signal subsequent divergence in efficacy (43), indicating that nutritional risk has not only baseline identification value but also a dynamic evolutionary component. Consistently, improvements in NLR, SII, PNI, and LIPI during treatment are often accompanied by better disease control and longer progression-free survival (43), indicating that immune-inflammatory and nutritional status are not static background factors but host signals directly involved in shaping treatment outcomes. In locally advanced disease, inflammatory-nutritional scores combined with persistent post-treatment inflammatory status can also stratify survival after consolidative immunotherapy (44), further suggesting that inflammatory-nutritional information has potential utility in front-loaded treatment decision-making. Notably, systemic inflammation may simultaneously relate to both toxicity risk and poor prognosis; pretreatment CRP elevation has been associated with a higher incidence of immune-related adverse events and reduced survival (45), suggesting that patients with high inflammatory burden may face a dual risk of limited benefit and increased treatment cost. Composite indicators based on early on-treatment changes in CRP and NLR can identify patients more likely to derive durable benefit at an early stage (46), and real-world data also indicate that SII and NLR are associated with prognosis while PNI is associated with treatment response (47). Therefore, integrating immune-inflammatory signals with body composition and nutritional variables is not simply a methodological extension, but a necessary step toward a model that more closely reflects the true pathophysiologic structure of nutritional risk in NSCLC. In essence, it advances the concept of nutritional risk from static malnutrition classification toward integrated recognition of host inflammatory-metabolic imbalance and immune-response potential.

Against the backdrop of previous evidence, the present study further indicates that estimating nutritional risk using body weight or BMI as surrogates for body composition and immune-inflammatory information may underestimate the true heterogeneity of host status in NSCLC. BMI is fundamentally a crude descriptor of body habitus; it cannot distinguish the relative contributions of muscle and fat, nor can it capture inflammation-driven lipid mobilization, muscle depletion, or impaired metabolic adaptation. It is therefore unsurprising that its association with treatment outcomes has been inconsistent or even contradictory across studies (48–50). In our study, BMI lost independent predictive value once body composition and immune-inflammatory variables were included, whereas skeletal muscle index, intermuscular adipose tissue volume, and inflammatory-nutritional indicators remained stable predictors. This suggests that conventional anthropometric measures capture only a superficial projection of nutritional vulnerability, whereas the host phenotypic structure jointly formed by muscle reserve, fat distribution, and inflammatory burden is what truly carries risk-stratification value. More importantly, our findings support not only baseline risk identification but also the notion that nutritional risk is dynamically evolving. Previous studies have shown that longitudinal changes in muscle and subcutaneous fat during treatment are more predictive of survival than a single baseline measurement (51, 52), indicating that nutritional risk is not a fixed label but a process state that continues to be updated under treatment stress, inflammatory fluctuation, and metabolic remodeling. Accordingly, the more important potential value of the fusion model developed here lies not merely in generating a one-time prediction, but in providing a scalable structural basis for future dynamic reassessment. This is especially important for clinical translation, because the ultimate purpose of nutritional risk stratification is not to add another prognostic marker, but to identify those patients who may derive the greatest benefit from earlier supportive care. Existing intervention studies have suggested that perioperative immunonutrition may improve postoperative nutritional and inflammatory status (53), while nutritional counseling combined with exercise and specific nutritional supplementation is feasible but shows variable efficacy across individuals (54); modulation of the microbiome and multimodal prehabilitation may also represent actionable pathways for improving host status (55–57). In parallel, perioperative changes in PNI and the combined phenotype of sarcopenia plus impaired immunonutritional status have been shown to carry stronger prognostic value (58, 59), and opportunistic chest CT-based frameworks for assessing respiratory muscle loss further reinforce the practical feasibility of thoracic imaging in risk assessment (60). The significance of our study therefore does not lie in merely repeating the known value of body composition or inflammatory indicators, but in proposing a more clinically realistic integrative paradigm: using routine chest CT as the entry point, opportunistic body composition measurement is transformed, together with nutritional and immune-inflammatory signals, into risk information that can support early identification, dynamic monitoring, and targeted intervention allocation. In doing so, it provides a stronger methodological basis for moving NSCLC nutritional management from experience-based judgment toward phenotype-driven care.

Several limitations of this study should be considered. First, this was a single-center retrospective cohort study. Although temporal validation, internal correction, and multiple sensitivity analyses were used to minimize overfitting and information leakage, residual confounding, selection bias, and clinical decision bias inherent to observational research cannot be completely excluded. In particular, under the highly heterogeneous treatment landscape of NSCLC, host states associated with different disease stages, treatment pathways, and intensities of supportive care are not fully exchangeable, which means that some of the risk signal captured by the model may still partly reflect treatment selection and clinical management strategies themselves. Second, although automated AI-based segmentation markedly improved standardization and reproducibility of body composition extraction, CT body composition phenotypes remain susceptible to differences in scan parameters, reconstruction algorithm, slice thickness, contrast status, and equipment. In other words, automation does not guarantee native transportability, and without cross-platform harmonization, unified calibration, and prospective quality-control workflows, the robustness of the model in real-world multicenter deployment may still be constrained by technical heterogeneity. Third, although peripheral blood immune-inflammatory indicators are easy to obtain and highly feasible in clinical practice, they are relatively non-specific biologically and can be influenced by infection, comorbidities, corticosteroid use, and antimicrobial treatment. In this study, they are therefore better interpreted as proximal indicators of host status rather than as mechanistically specific biomarkers. Future studies should validate the model in multicenter prospective cohorts and incorporate longitudinal CT body composition trajectories, dynamic immune-inflammatory signals, and patient-reported outcomes into joint modeling in order to more fully characterize the evolution of nutritional vulnerability. More importantly, interventional studies centered on nutritional support, exercise intervention, or immunonutritional strategies are needed to determine whether the high-risk phenotype defined here is modifiable, and whether model-guided stratified management can yield quantifiable clinical net benefit, thereby moving this framework from a predictive tool toward a decision-guiding clinical pathway capable of improving outcomes.

## 5 Conclusion

Based on body composition phenotypes derived from routine chest CT, combined with clinical nutritional indicators and immune-inflammatory signals, we developed and validated a multidimensional nutritional risk prediction model for risk stratification in patients with NSCLC. The results indicate that the fusion model can provide refined prediction of nutrition-related adverse clinical trajectories and long-term survival outcomes before treatment and during the early treatment phase, while maintaining stable performance across multiple model comparisons and sensitivity analyses. Compared with traditional approaches based primarily on body weight or a single nutritional assessment tool, this method more comprehensively integrates host biological characteristics reflected by muscle reserve, fat distribution, and inflammatory status, and therefore more closely approximates the true structure of nutritional vulnerability.

From a translational clinical perspective, the integrated framework proposed in this study uses routine imaging as the entry point and combines it with readily available laboratory indices to provide a scalable tool for early risk identification in patients with NSCLC. Beyond improving nutritional risk stratification, the model offers a quantitative basis for forward placement of intervention and for individualized supportive treatment planning. Further multicenter prospective studies and interventional trials are warranted to validate its external applicability and clinical net benefit, and to promote the transition of nutritional management based on body composition and immune-inflammatory phenotypes from risk assessment to precision intervention.

## Data Availability

The raw data supporting the conclusions of this article will be made available by the authors, without undue reservation.
