# Supplementary material for: Multidimensional modeling and stratification of nutritional risk in non-small cell lung cancer based on artificial intelligence–derived CT body composition phenotypes
Source: Front Nutr. 2026 Aug 20;13:1842431. doi: 10.3389/fnut.2026.1842431 (PMC13537986; doi:10.3389/fnut.2026.1842431)
Supplement: Supplementary file 1 [file Table_1.docx]

**Supplementary Figure S1. Evaluation of segmentation performance and agreement of the artificial intelligence body composition analysis model.**

*(A) Distribution of Dice similarity coefficients between automatic segmentation and manual annotation for skeletal muscle, subcutaneous adipose tissue, and visceral adipose tissue.
(B) Comparison of Dice similarity coefficients across tissue types between the development cohort and the temporal validation cohort, used to assess the stability of segmentation performance across different temporal datasets.*

*(C) Agreement analysis between automated and manual measurements, showing the correlations and regression relationships for the three categories of body composition parameters.*

**Supplementary Figure S2. Restricted cubic spline analyses of the associations between inflammation-related markers and the primary endpoint.**

*(A) Association between log(CRP) and the short-term adverse nutrition-related clinical trajectory, showing a mildly nonlinear trend.*

*(B) Association between log(NLR) and the primary endpoint, showing an overall approximately linear pattern.*

*(C) Association between log(PLR) and the primary endpoint, with no significant nonlinear feature observed.*

*(D) Association between log(SII) and the primary endpoint, showing a relatively more pronounced nonlinear trend.*

# Table S1. Components of the composite endpoint and GLIM-stratified comparisons

| Variable | Overall (n=656) | Non-malnutrition (n=418) | Malnutrition (n=238) | P value |
| --- | --- | --- | --- | --- |
| Composite endpoint components |  |  |  |  |
| Treatment delay/modification/interruption, n (%) | 91 (13.9) | 45 (10.8) | 46 (19.3) | 0.003 |
| Unplanned hospitalization, n (%) | 61 (9.3) | 29 (6.9) | 32 (13.4) | 0.006 |
| ≥Grade 3 adverse events, n (%) | 48 (7.3) | 20 (4.8) | 28 (11.8) | 0.001 |
| Postoperative complications, n (%) | 33 (5.0) | 14 (3.3) | 19 (8.0) | 0.009 |
| 90-day mortality, n (%) | 18 (2.7) | 6 (1.4) | 12 (5.0) | 0.012 |
| Clinical characteristics |  |  |  |  |
| Age, years | 64 (57–71) | 62 (55–69) | 67 (60–73) | <0.001 |
| ECOG PS ≥2, n (%) | 187 (28.5) | 90 (21.5) | 97 (40.8) | <0.001 |
| Stage III–IV, n (%) | 422 (64.3) | 247 (59.1) | 175 (73.5) | 0.002 |
| Nutritional variables |  |  |  |  |
| BMI, kg/m² | 22.3 (20.1–24.8) | 23.1 (21.0–25.4) | 20.9 (18.7–23.1) | <0.001 |
| Weight loss (%), 6 months | 6.8 (3.2–11.5) | 4.1 (1.8–7.2) | 9.8 (6.4–14.3) | <0.001 |
| Albumin, g/L | 37.2 ± 5.1 | 39.2 ± 4.6 | 34.1 ± 4.8 | <0.001 |
| PNI | 45.1 ± 6.8 | 48.1 ± 6.3 | 40.2 ± 6.1 | <0.001 |
| Inflammatory markers |  |  |  |  |
| NLR | 3.6 (2.2–5.4) | 2.9 (1.9–4.3) | 4.8 (3.1–6.9) | <0.001 |
| SII | 875 (560–1320) | 720 (480–1050) | 1180 (820–1680) | <0.001 |
| Imaging parameters |  |  |  |  |
| SMI, cm²/m² | 42.6 ± 8.7 | 44.8 ± 8.2 | 38.9 ± 7.5 | <0.001 |
| Muscle attenuation, HU | 36.5 ± 7.2 | 38.6 ± 6.9 | 33.2 ± 6.8 | <0.001 |
| VAT index, cm²/m² | 38.2 (24.5–56.8) | 34.6 (22.1–52.3) | 45.1 (28.7–63.9) | 0.004 |
| IMAT volume, cm³ | 210 (150–295) | 185 (132–260) | 248 (182–330) | 0.002 |

# Table S2. Correlation, Collinearity Assessment, and Selection of Candidate Imaging Predictors

| Variable | Correlation (r) | VIF | Univariable OR (95% CI) | P value | Final decision |
| --- | --- | --- | --- | --- | --- |
| Skeletal muscle area | 0.78 | 4.92 | 0.94 (0.89–0.98) | 0.011 | Excluded |
| Skeletal muscle volume | 0.82 | 5.36 | 0.93 (0.89–0.98) | 0.006 | Excluded |
| Skeletal muscle index | 0.82 | 3.14 | 0.90 (0.85–0.95) | <0.001 | Retained |
| Skeletal muscle attenuation | 0.41 | 2.27 | 0.88 (0.83–0.94) | <0.001 | Retained |
| Subcutaneous fat area | 0.76 | 4.21 | 1.04 (0.99–1.09) | 0.127 | Excluded |
| Subcutaneous fat volume | 0.79 | 4.68 | 1.05 (1.00–1.10) | 0.064 | Excluded |
| Visceral fat area | 0.79 | 4.87 | 1.09 (1.03–1.16) | 0.004 | Excluded |
| Visceral fat index | 0.79 | 3.48 | 1.12 (1.05–1.19) | 0.001 | Retained |
| Intermuscular fat area | 0.74 | 4.56 | 1.11 (1.04–1.18) | 0.002 | Excluded |
| Intermuscular fat volume | 0.77 | 3.62 | 1.15 (1.08–1.22) | <0.001 | Retained |
| Muscle-to-fat ratio | 0.69 | 5.11 | 0.91 (0.86–0.97) | 0.005 | Excluded |
| Visceral-to-subcutaneous fat ratio | 0.66 | 4.74 | 1.07 (1.01–1.13) | 0.018 | Excluded |

Abbreviations: VIF, variance inflation factor; OR, odds ratio; CI, confidence interval. Univariable ORs were calculated per standardized unit increase. Variable selection was based on clinical relevance, collinearity diagnostics, and penalized regression.

Table S3. Incremental Predictive Value of AI-derived Body Composition Features (Temporal Validation Cohort)

| Metric | Estimate | 95% CI | P value | Interpretation |
| --- | --- | --- | --- | --- |
| Net Reclassification Improvement (overall) | 0.186 | 0.061–0.311 | 0.004 | Significant improvement in overall risk reclassification |
| Event NRI | 0.124 | 0.038–0.210 | 0.006 | Higher proportion of events correctly up-classified |
| Non-event NRI | 0.062 | 0.012–0.112 | 0.015 | Reduction in unnecessary high-risk classification |
| Integrated Discrimination Improvement (IDI) | 0.072 | 0.019–0.125 | 0.008 | Improved separation of predicted probabilities |
| Change in C-index | 0.082 | 0.041–0.123 | <0.001 | Consistent improvement in discrimination |
| Mean predicted risk (events) | +0.067 | 0.031–0.103 | <0.001 | Increased predicted risk among events |
| Mean predicted risk (non-events) | -0.028 | -0.052 to -0.004 | 0.021 | Decreased predicted risk among non-events |
| Category-free NRI | 0.179 | 0.055–0.303 | 0.005 | Robust improvement without predefined categories |

Abbreviations: NRI, net reclassification improvement; IDI, integrated discrimination improvement; CI, confidence interval.

Table S4. Comprehensive Sensitivity Analyses of the Nutrition Risk Model

| Analysis / Variable | Primary analysis | NRS-2002 endpoint | Restricted cohort | 3D model | Multiple imputation |
| --- | --- | --- | --- | --- | --- |
| — Model performance — |  |  |  |  |  |
| AUC (Development) | 0.842 | 0.829 | 0.836 | 0.854 | 0.840 |
| AUC (Validation) | 0.816 | 0.801 | — | — | 0.813 |
| Calibration slope | 0.97 | — | 0.96 | — | 0.96 |
| Brier score | 0.167 | — | 0.169 | — | 0.168 |
| — Key predictors stability (OR) — |  |  |  |  |  |
| Weight loss (%) | 1.24 | 1.21 | 1.23 | 1.25 | 1.23 |
| PNI | 0.95 | 0.96 | 0.95 | 0.94 | 0.95 |
| log(SII) | 1.18 | 1.16 | 1.17 | 1.19 | 1.18 |
| SMI | 0.91 | 0.92 | 0.90 | 0.89 | 0.91 |
| Intermuscular fat | 1.14 | 1.12 | 1.13 | 1.16 | 1.14 |
| — Classification consistency — |  |  |  |  |  |
| Agreement rate | 0.91 | — | — | — | — |
| Kappa | 0.82 | — | — | — | — |

Abbreviations: AUC, area under the curve; PNI, prognostic nutritional index; SII, systemic immune-inflammation index; SMI, skeletal muscle index.

Table S5. Subgroup Analyses of the Nutrition Risk Model Performance

| Subgroup | n | AUC | 95% CI | P for interaction | Interpretation |
| --- | --- | --- | --- | --- | --- |
| — Age — |  |  |  |  |  |
| <65 years | 248 | 0.832 | 0.781–0.883 | 0.214 | Consistent performance |
| ≥65 years | 184 | 0.818 | 0.759–0.877 |  |  |
| — Sex — |  |  |  |  |  |
| Male | 276 | 0.840 | 0.792–0.888 | 0.327 | No significant interaction |
| Female | 156 | 0.809 | 0.742–0.876 |  |  |
| — Tumor stage — |  |  |  |  |  |
| Stage I–II | 162 | 0.861 | 0.802–0.920 | 0.156 | Stable across stages |
| Stage III–IV | 270 | 0.802 | 0.748–0.856 |  |  |
| — Treatment — |  |  |  |  |  |
| Systemic therapy | 298 | 0.845 | 0.798–0.892 | 0.273 | Robust in treated patients |
| No systemic therapy | 134 | 0.804 | 0.735–0.873 |  |  |
| — Nutritional status — |  |  |  |  |  |
| Low risk (baseline) | 210 | 0.826 | 0.774–0.878 | 0.198 | Stable across baseline nutrition |
| High risk (baseline) | 222 | 0.834 | 0.781–0.887 |  |  |

Abbreviations: AUC, area under the curve; CI, confidence interval.
